# Becoming a fighter: phenomenological dimensions of personal development in beginner martial arts and combat sports practitioners

**DOI:** 10.3389/fpsyg.2026.1910206

**Published:** 2026-09-18

**Authors:** Yan Lázaro Santos, Carlos Gutiérrez-García, Cristiano Roque Antunes Barreira

**Affiliations:** 1 Faculdade de Filosofia, Ciências e Letras, Universidade de São Paulo (USP), Ribeirão Preto, Brazil; 2 Departamento de Educación Física y Deportiva, Facultad de Ciencias de la Actividad Física y del Deporte, Universidad de León, León, Spain; 3 Escola de Esportes e Educação Física de Ribeirão Preto, Universidade de São Paulo (USP), Ribeirão Preto, Brazil

**Keywords:** combat sports, martial arts, personal development, phenomenology, sport psychology

## Abstract

**Introduction:**

Martial arts and combat sports (MA&CS) place practitioners in bodily and relational situations close to violence while being organized through the reciprocal structure of corporal fight. Understanding personal development in these practices requires attention to how practitioners experience, sustain, and respond to combative engagement. Disruptive situations are moments in which this structure is strained, exposing the limits of practitioners’ resources without necessarily producing development. The aim of this study is to identify and comprehend how adult beginner MA&CS practitioners experience processes of personal development in relation to disruptive situations encountered during practice.

**Method:**

The study follows an empirical-phenomenological approach within a cross-national framework. Seventeen retrospective semi-structured interviews with beginner practitioners were selected from a database of 157 interviews conducted in Brazil and Spain. Analysis involved psychological, eidetic, and intersubjective reductions, supported by suspensive listening and intentional crossing.

**Results:**

The findings were organized into three interdependent dimensions. The Psychic dimension concerns the tension inherent to confronting an opponent, including fear, frustration, uncertainty, and difficulties remaining engaged. The Moral/Spiritual dimension concerns sensitivity to the partner-opponent’s integrity and the regulation of force, intensity, and conduct required to preserve the reciprocal encounter. The Corporal/Technical dimension concerns the incorporation of embodied possibilities for attacking, defending, adjusting distance, and responding under pressure. Because corporal fight is lived through bodily engagement, this dimension provides the concrete medium through which psychic tension and Moral/Spiritual positioning are enacted.

**Discussion:**

When development is described, it appears as a progressive reorganization of these dimensions and an expanded capacity to enter, respond to, and sustain corporal fight. Participants also report avoidance, emotional disorganization, excessive force, injury, and withdrawal, indicating that disruptive experiences are not inherently developmental. Competition intensifies these demands, while developmental possibilities depend on practitioners’ resources and on pedagogical and relational conditions, including instructor guidance, peer conduct, partner selection, and regulation of training intensity.

## 1 Introduction

Martial arts and combat sports (MA&CS) have attracted growing interest in sport psychology because of their potential contributions to personal development, emotional regulation, and social learning. Reviews suggest that MA&CS may promote positive outcomes among young practitioners, including resilience, prosocial behavior, and other psychosocial benefits (Kooi, 2020; Theeboom, 2012; Chen et al., 2020). However, the evidence remains limited (Lafuente et al., 2021) and inconsistent, with some studies suggesting that combative experiences may also increase aggressiveness (Meller, 2020).

To examine this inconsistency, sociological and ethnographic studies have described learning to fight as a gradual transformation of bodily dispositions, perceptual orientations, and practical knowledge that shapes participation in combat situations (Wacquant, 2006; Sánchez García, 2008). These studies suggest that development in combat practices cannot be understood apart from the bodily and relational dynamics that structure fighting.

Nevertheless, research on development in MA&CS has focused mainly on psychological variables and socialization processes, often overlooking how practitioners experience the combat situation itself. Phenomenological approaches emphasize that sporting practices are better understood through lived corporeality and athletes’ intentional engagement with situations of action (Allen-Collinson, 2009, 2011). Recent studies have begun to describe the experiential structure of fighting by distinguishing forms of combat and examining how practitioners enter and sustain the intentional orientation characteristic of combat situations (Barreira, 2017a; Telles et al., 2018; Barreira et al., 2025). However, little is known about how beginner practitioners experience the practical, bodily, and relational demands of learning to fight, particularly in situations that challenge their capacity to sustain corporal fight.

Addressing this gap requires understanding learning to fight as more than the acquisition of techniques or the internalization of norms. Rather, it involves developing the capacity to sustain a sporting form of bodily and relational engagement with an opponent. Corporal fight is organized as a mutually sustained interaction that requires tension, control, and recognition of the other as a partner in the fight (Barreira et al., 2025). The maintenance of this relational structure distinguishes corporal fight from hostile aggression, even when both involve similar bodily actions.

These experiential distinctions are systematized in the *Theory of the Phenomenon of Bodily Combat*^1^ (TPBC), an empirical-phenomenological framework developed to conceptualize how bodily combat is lived and intersubjectively organized in MA&CS (Barreira, 2010, 2013, 2017a, 2019b, 2019a; Miranda and Barreira, 2022; Coelho and Barreira, 2020; Barreira et al., 2025). Rather than classifying combative actions according to their external form, the TPBC schematizes six combative manifestations—corporal fight, brawl, self-defense, combat play, duel, and instrumental offensive combat—according to three interrelated features: the intention motivating the encounter, the degree to which each participant remains open to the other as a subject, and whether their intentions are reciprocally or unilaterally organized (Barreira, 2019a; Miranda and Barreira, 2022). These schemes therefore describe distinct experiential structures of bodily combat (Barreira et al., 2025).

Of particular relevance to MA&CS is corporal fight, the TPBC manifestation that structures most training and competitive encounters. In corporal fight, both participants reciprocally seek to overcome one another while continuing to recognize the opponent as another subject who jointly sustains the fight; victory, rather than the negation of the other, organizes the interaction (Barreira, 2017a, 2019a; Barreira et al., 2025). By contrast, brawl is structured by hostility and by a closure toward the other, who ceases to be experienced as a partner-opponent and becomes the object of hostile action (Barreira, 2019b, 2019a). This distinction provides the conceptual basis for the present study, in which disruptive experiences become the analytical focus.

To clarify the terminology adopted throughout the manuscript, Table 1 distinguishes the broader field of practices investigated from the phenomenological categories derived from the TPBC.

**Table 1:** Terminological distinctions adopted in the manuscript.

Term	Definition and use in this study within the theoretical framework of the TPBC
Martial arts	Refers specifically to practices or literature identified as martial arts.
Combat sports	Refers specifically to competitive combative modalities or literature focused on combat sports.
MA&CS	Umbrella term used to designate martial arts and combat sports included in the study.
Bodily combat	Broad phenomenological domain theorized by the TPBC, encompassing distinct intentional and intersubjective manifestations of combat beyond sporting contexts.
Corporal fight or fight	Specific manifestation of bodily combat central to this study, in which participants reciprocally seek to overcome one another while continuing to recognize the other as a partner-opponent; within the TPBC, corporal fight corresponds to the sporting form of combat.
Brawl	Manifestation of bodily combat structured by hostility and closure toward the other.

These terms are not treated as mutually exclusive categories, as some disciplines may be approached as martial arts in relation to their historical, cultural, and pedagogical traditions and as combat sports when organized within institutionalized sporting and competitive settings.

In contemporary scientific literature, disruptive experiences refer to events that challenge established expectations, understandings, or intentions and require the reorganization of existing schemas. A disjunction between expectation and experience may prompt accommodation — that is, the modification of intentional schemas to resolve the conflict (Gillespie, 2020; Estecha Querol et al., 2020). Disruption therefore marks a threshold at which established ways of understanding and acting become insufficient, creating a potential, rather than automatic, opportunity for learning (Gillespie, 2020).

Within MA&CS, disruption describes borderline moments in which practitioners approach the limits of corporal fight and their conduct may shift from disciplined fighting toward brawl (Santos et al., 2025). Whether such moments support development may depend on structured and ethically responsible training environments, including instructor guidance, partner selection, peer conduct, and regulation of training intensity (Ciaccioni et al., 2025a, 2026). These events provide a distinctive analytical window because they reveal the conditions required to sustain corporal fight precisely when practitioners’ resources are strained. They may expose difficulties in managing combative tension, regulating force, responding technically, or recognizing the other as a partner-opponent. Disruptive experiences therefore offer access to the lived process of personal development without implying that disruption is inherently developmental.

The initial stages of training are particularly relevant in this regard. Beginners may not yet have developed the technical, psychological, and relational resources needed to manage combative tension and respond to fear, pain, uncertainty, emerging hostility, or moral tensions involving training partners. Situations that challenge their expectations may therefore expose the limits of their capacity to sustain the sporting structure of the encounter. Sociological analyses of combat-sport training suggest that moments of rupture—such as receiving a punch for the first time—can substantially alter practitioners’ engagement with the practice (Sánchez García, 2008). However, such experiences have rarely been examined phenomenologically from the perspective of how practitioners themselves live through and interpret them.

Previous TPBC studies have described the intersubjective structures through which different combative manifestations are constituted (Barreira, 2017a, 2019a; Miranda and Barreira, 2022; Barreira et al., 2025). What remains less understood is the lived process through which practitioners progressively become able to inhabit and sustain corporal fight. Accordingly, the present study examines how beginner MA&CS practitioners experience disruptive situations and how these experiences relate to the lived process of personal development within practice.

## 2 Objective

The aim of this study is to identify and comprehend how adult beginner MA&CS practitioners experience processes of personal development in relation to disruptive situations encountered during practice.

## 3 Materials and methods

This study employed an observational, theoretical and descriptive design, using qualitative data gathered through retrospective semi-structured interviews. It was conducted within the framework of a broader research project (FAPESP No. 2019/11527–6) through an empirical–phenomenological lens, with the objective of providing a deeper understanding of practitioners’ lived experiences (Valério and Barreira, 2015). The protocol for the study was approved by the local research ethics committees of the Universidade de São Paulo, Brazil (code: 57691616.0.0000.5659) and the Universidad de León, Spain (ETICA-ULE-076-2024).

### 3.1 Collective framework

This study forms part of a long-term collective research project aimed at developing a psychology of martial arts and combat sports (MA&CS) grounded in practitioners’ lived experiences, guided by a phenomenological perspective (FAPESP-funded project No. 2019/11527–6). Practitioners in the broader database were recruited through a non-probability strategy combining direct outreach to MA&CS organizations with snowball sampling. The researchers contacted clubs, training centers, and instructors to obtain permission to disseminate the study invitation among adult volunteers. Instructors and participating practitioners were also invited to refer other potential participants. Training sites were not selected according to predefined criteria concerning competitive level, institutional profile, or practitioner expertise; rather, they entered the recruitment network through initial contacts and subsequent referrals.

The study drew on a cross-national database comprising 157 semi-structured interviews conducted in Brazil (*n* = 133) and Spain (*n* = 24). To support the collective objective of developing a psychology of MA&CS grounded in lived experience (Barreira et al., 2025), participant recruitment in both countries was conducted without prior expertise-based selection. The design was not intended as a systematic comparison between national cultures.

Methodological rigor was supported through shared researcher training, systematic peer debriefing, and collaborative analysis (Ahmed, 2024). The research team comprised five researchers who received methodological training under the supervision of the senior author. During this process, the team collectively examined interview transcripts and psychological reductions to establish a shared phenomenological approach. Researchers contributed to the transcription and psychological reduction of interviews beyond those directly related to their individual projects and identified accounts potentially relevant to other investigations within the collective framework. These procedures supported methodological consistency and peer discussion during the initial analytical stage. However, the researcher responsible for each study retained final responsibility for participant selection and for the subsequent eidetic and transcendental—or intersubjective—reductions. Similar applications of this framework are described by Barreira et al. (2025) and Santos et al. (2025).

### 3.2 Participants

The study included 17 adult beginner MA&CS practitioners (7 women and 10 men), aged between 19 and 61 years (M = 31.4, SD = 12.6). Participants were eligible for the present study when they: (1) are 18 years of age or older; (2) actively practice one or more of the eight MA&CS disciplines included in the database at the time of the interview; and (3) are classified as beginners according to their experiential relationship with concrete combative situations.

Potential cases were initially screened among practitioners with less than 3 years of training. Training duration functioned only as a preliminary criterion because formal rank and time in practice do not necessarily correspond to familiarity with combative situations across different MA&CS disciplines. The first author subsequently reviewed the interviews using an experiential criterion focused on whether practitioners are still developing the capacity to enter, respond to, and sustain corporal fights.

This classification considered the overall configuration of each participant’s account rather than any single reaction or predetermined indicator. Fear, intimidation, uncertainty, mental blockage, technical difficulties, and avoidance of sparring or competition were treated as possible manifestations of limited familiarity with combative situations, but none of these manifestations is required for inclusion. Practitioners were classified as beginners when their accounts indicate that they are still learning to manage the practical demands of fighting (Barreira et al., 2025; Santos et al., 2025).

Whereas cognitive approaches commonly distinguish beginner and experienced practitioners according to expertise or skill level (Zhang et al., 2022), the present framework examined how practitioners describe and respond to concrete combative situations. This approach allowed beginner status to be identified through the practitioner’s lived relationship with combative pressure and the practical demands of fighting (Barreira et al., 2025; Barreira and Coelho Júnior, 2023; Barreira, 2017d; Barreira and Ranieri, 2013). The first author performed the final classification and selected the 17 interviews included in the study. Interpretative questions may be discussed within the collective research framework, but final decisions regarding participant inclusion remain the responsibility of the first author.

All participants received information about the aims and procedures of the study and provide written informed consent before participation. Interviews were audio-recorded only with participants’ authorization. All identifying information was removed from the dataset, and pseudonyms inspired by characters from animated productions directly or indirectly related to MA&CS were used throughout the study, as presented in Table 2.

**Table 2:** Participant’s description.

Nickname	Modality	Age	Sex	Time of practice	Location	Degree
Lee	Taekwondo	58	M	8 months	CE-BR	Yellow belt
Katsumi	Judo	40	M	8 months	MG-BR	White belt
Diane	Capoeira Angola	24	F	3 years and 6 months	SP	D/A
Toph	Judo	42	F	2 years	MG-BR	Blue belt
Mikasa	Boxe	23	F	1 year	MT-BR	Amateur
Doppo	Karate	61	M	4 years and 6 months	GO-BR	Purple belt
Alvida	Taekwondo	19	F	1 year	MG-BR	Yellow belt
Yancha	Brazilian Jiu-jitsu	24	M	3 years	PR-BR	Blue belt
Ichigo	Judo	21	M	3 years and 6 months	CE-BR	Orange belt
Karla	Brazilian Jiu-jitsu	34	F	3 years	PR-BR	Purple belt
Katara	Brazilian Jiu-jitsu	26	F	3 years	MG-BR	Blue belt
Kuririn	Brazilian Jiu-jitsu	23	M	1 year and 6 months	Spain	White belt
Canary	Boxe	26	F	1 year and 2 months	Spain	D/A
Choji	Brazilian Jiu-jitsu	27	M	8 months	Spain	White belt
Edward	Brazilian Jiu-jitsu	37	M	2 years	Spain	White belt
Hatsumi	Brazilian Jiu-jitsu	21	M	2 months	Spain	White belt
Belmont	Kick-Boxing; Brazilian Jiu-jitsu; MMA	27	M	8 months	Spain	White belt(BJJ); D/A (K. B); Amateur (MMA)

Abbreviations refer to Brazilian states, grouped within the country’s geographic regions. CE-BR = Ceará (Northeast); MG-BR = Minas Gerais (Southeast); SP = São Paulo (Southeast); MT-BR = Mato Grosso (Central-West); GO-BR = Goiás (Central-West); PR-BR = Paraná (South).

### 3.3 Instruments

The primary instrument used in this study was a semi-structured interview guide developed collectively by the members of the *Research Group Phenomenology and Bodily Experience* (GFEPAC, Brazilian acronym) to elicit detailed descriptions of lived experience, following a Husserlian empirical-phenomenological framework. The guide was designed to operationalize a shared attentional and methodological effort to delimit the lived experiences relevant to the object of inquiry and to elicit detailed first-person descriptions of concrete (Barreira, 2017b).

The interview guide comprised open-ended prompts concerning disruptive, difficult, tense, or conflict-laden situations in martial arts and combat sports, followed by exploratory questions intended to clarify how participants had perceived, experienced, and retrospectively evaluated those events. Examples of guiding questions included: “Describe a situation during practice that felt delicate, difficult, tense, or conflict-ridden” and “Reflecting on it now, how do you evaluate that experience? Have you encountered similar situations since then?” Examples of disruptive situations included concrete episodes involving intimidation, loss of control, escalating force, interpersonal conflict, injury, or difficulty sustaining sparring - e.g., a situation in which Canary observes a fellow competitor losing their temper during training and shouting; when a practitioner becomes angry and develops a primary intention to hurt the opponent; or when they experience fear to the point of needing to withdraw from the fight. The complete interview guide is provided in the Supplementary materials.

### 3.4 Procedures

Interview conduction constituted the empirical stage of this empirical-phenomenological design. All interviews were conducted individually, face-to-face, and audio-recorded, with durations ranging from 30 to 75 min. Prior to participation, all participants provided written informed consent.

As explained above, the interviews followed a semi-structured guide. Although guided by common thematic axes, the interview remained flexible, allowing follow-up questions to explore aspects that emerged from each participant’s account. Questions were formulated to encourage descriptions of concrete experiences rather than opinions, explanations, or theoretical interpretations.

Both the design of the guide and the conduction of the interviews followed phenomenological methodological procedures, particularly Suspensive Listening (Barreira, 2018; Barreira and Coelho Júnior, 2023; Barreira et al., 2025; Santos et al., 2025). This stance involved maintaining openness toward the participant’s experience while suspending prior assumptions, explanatory frameworks, and evaluative judgments. Throughout the interview, the researcher sought to facilitate the expression of first-person experience and clarify experiential meanings through descriptive exploration rather than interpretative guidance (Ranieri and Barreira, 2010; Barreira and Ranieri, 2013; Valério and Barreira, 2015). This procedure aimed to support access to the phenomenon as it was lived and described by the participant.

### 3.5 Data analysis

Interviews were audio-recorded, transcribed verbatim, and reviewed line-by-line by the researchers. Data are analyzed using the empirical-phenomenological method (Barreira, 2017d; Barreira et al., 2025; Santos et al., 2025), which involves three successive analytical movements: psychological, eidetic, and transcendental (or intersubjective) reductions.

The analysis began with psychological reduction, in which each interview was examined individually to identify how the investigated phenomenon was experienced and described by the participant. Subsequently, through eidetic reduction, descriptions were systematically compared across participants to identify convergences, divergences, and recurring experiential patterns. At this stage, intentional crossing functioned as an operative methodological resource. Like Suspensive Listening, it oriented the researcher’s intentional stance and supported the phenomenological transition from individual descriptions to eidetic clarification. More specifically, intentional crossing involved the systematic comparison of the intentional structures expressed across the accounts. It was used to identify experiential features that remained recognizable across variations in participants, MA&CS disciplines, and contexts, while divergent accounts were examined to refine the scope of each proposed dimension. Finally, transcendental—or intersubjective—reduction sought to clarify the intentional and intersubjective structures underlying these recurring patterns, allowing the development of a phenomenological description grounded in shared modes of experience rather than empirical frequency or prior theoretical assumptions (Ales Bello, 1998, 2006; Barreira, 2017d).

To enhance credibility, dependability, and confirmability, the analysis is conducted within the Collective Framework described above. Transcriptions, participant identification, and psychological reductions are collaboratively reviewed by trained researchers, with experiential descriptions being discussed and validated throughout the analytical process. This procedure provides continuous peer debriefing, minimizes individual interpretative bias, and maintains a transparent audit trail linking analytical decisions to participants’ accounts (Ahmed, 2024; Barreira et al., 2025; Santos et al., 2025).

Following this phenomenological approach, the results are organized into clusters of meaning, referred to in this study as dimensions. The identification of a dimension is not based on the numerical frequency of specific expressions or on a predetermined coding threshold. During the eidetic reduction, experiential descriptions are compared across interviews to examine convergences, variations, and divergences. A dimension is retained when a recurring experiential structure is supported by concrete first-person accounts, remains recognizable across different participants and combative contexts, and contributes distinctly to explaining the investigated phenomenon. Contrasting accounts are also examined to refine the boundaries and meaning of each dimension. The resulting dimensions are understood as interdependent analytical structures rather than mutually exclusive categories.

## 4 Results

The results are organized into three comprehensive and interrelated dimensions, descriptive of the experience of personal development of beginner practitioners of 08 modalities of MA&CS through disruptive situations during their practice: Psychic Dimension; Moral/Spiritual dimension and Corporal/Technical Dimension.

### 4.1 Psychic dimension

Across participants’ accounts, combative tension is present even in the absence of explicit conflict or hostility. Participants describe fighting itself as an intersubjective situation marked by psychic tension, without necessarily involving the objectification of the other or a shift toward brawl. Violence appears as an extreme possibility of this tension rather than as a condition for its emergence. The tension is therefore experienced within the confrontation itself, before any explicit rupture of the reciprocal structure of corporal fight.

**Interviewer:** And even without conflict, do you consider (combat) a situation of tension?

**Interviewee:** Yes. That’s true. After all, they are hitting you, and even if you have nothing against that person, receiving a blow can affect you a little. I think so. (Canary, Boxing, 26 years old, woman, our translation).

Canary explicitly describes this tension during sparring, the combative training situation in boxing. In her account, the possibility of being struck affects the practitioner even in the absence of personal hostility toward the opponent.

**Interviewee**: I would get a little frustrated. I would freeze up a bit and I think I reacted worse in training than I could have. Because of that, I would get frustrated for not being able to defend myself well and ending up taking the blow. That would make me freeze a little and, perhaps, not counterattack, I think. But more because of a (...) mental block. Because physically, I could not have done it any differently. (Canary, Boxing, 26 years old, woman, our translation).

Canary describes frustration, freezing, and difficulty responding after being struck. Rather than counterattacking, she remains defensively inhibited and characterizes her reaction as a “mental block.” She also retrospectively relates this restricted response to her limited capacity to act differently at that stage of practice. Belmont reports similar experiences in which technical unfamiliarity and limited habituation to combat constrain the practitioner’s response:

**Interviewee:** When you face someone much more skilled, you have no control over anything. The fight unfolds the way they want, the distance is the one they decide—you are at the mercy of the other. No one likes being at someone else’s mercy. And, on top of that, there is the fact of taking blows. When you are not used to taking punches to the face, the stress is even greater. (Belmont, BJJ/MMA/Kickboxing, 27 years old, man, our translation).

Participants also describe heightened pressure when facing substantially more experienced training partners. Hatsumi reports this experience after only 2 weeks of practice, when the force used by his sparring partner exceeded his expectations:

**Interviewer**: How was that for you?

**Interviewee**: Well. The first time, of course, it surprises you. Because you think: “Wow, I wasn’t expecting that.” But then, well, you become more attentive. Or you tell him: “Hey, take it easy a little,” or something like that.

[...]

**Interviewer**: And how did you evaluate the situation?

Interviewee: Nothing, man. I think it’s something common, something that can happen. (...) I mean, I did not take it personally, I did not get upset, or anything like that. (Hatsumi, BJJ/MMA, 21 years old, man, our translation).

Hatsumi describes initial surprise, followed by increased attention and a request that his partner moderate the intensity. He retrospectively presents the episode as a common occurrence within practice and does not interpret the partner’s conduct as personal hostility.

Choji describes a related form of tension at the beginning of his BJJ practice. His intimidation arises not from another practitioner’s deliberate conduct but from uncertainty regarding the demands of training, the people he would encounter, and the possibility of injury:

**Interviewee**: At the beginning, yes, but not because someone tried to intimidate me, but rather because of something more personal, related to my own insecurity. I arrived here, it was something new, and I did not know who I would encounter, whether they would hurt me a lot. So, at first, I felt a little intimidated for not knowing what to do, but beyond that, I did not have the feeling that anyone wanted to intimidate me or that I actually felt intimidated. (Choji, BJJ, 27 years old, man, our translation).

Choji locates this intimidation in his unfamiliarity with the training environment and his uncertainty about how to act. His statement that he did not know what to do also indicates a limited capacity to respond to the practical demands of the combative situation.

In a later episode, Choji describes reacting without deliberation while attempting to escape from a training partner:

**Interviewee**: Yes, not that I can recall. I think maybe the most would be the other day when I trained with you—we were there and I kind of shook you off like that [*interviewee imitates a violent shaking motion with both hands*]. I do not think I usually, like… lose control. (…) Because I think at that moment I wasn’t thinking; I just wanted to get away from you, you know? (…) I had to get out, and at that moment I did not think—I just shook you off, and that was it. (Choji, BJJ, 27 years old, man, our translation).

In this account, combative pressure is accompanied by an immediate and unplanned response. Choji contrasts acting deliberately with simply attempting to escape, describing a moment in which the urgency of leaving the position restricted his capacity to organize his actions.

Participants also describe competition as intensifying the tension of fighting. Canary distinguishes sparring with familiar teammates from facing an unknown opponent whose abilities and use of force cannot be anticipated:

**Interviewee**: In general, well, more in competition. Because it’s not the same thing to spar with your teammates, since, after all, you know they are not really going to hurt you. But stepping into the ring with someone unknown… there is always that fear: “What if they are much better than me and I come out of it badly?” So, I think mainly in competition, because the first time especially, I felt a lot of fear. (Canary, Boxing, 26 years old, woman, our translation).

Canary associates this fear with uncertainty about the opponent and the possibility of being harmed. After the event, however, she evaluates her performance positively: “But after it’s over, you think: ‘I did it, I acted well,’ and I think that increases self-confidence” (Canary, Boxing, 26 years old, woman, our translation).

Across these accounts, tension is associated with different responses. Participants describe freezing, uncertainty, defensive inhibition, heightened attention, requests to moderate intensity, and retrospective recognition of their own capacity to fight. Some accounts therefore include changes in how practitioners understand and respond to combative pressure, whereas others remain marked by restricted or disorganized action.

Canary also recounts an episode in which frustration with performance leads a practitioner to interrupt training. The participant involved was preparing for competition and expected to perform better during the session:

**Interviewee**: He thought he would be able to perform better and, that day, he could not, because he was tired. And he became completely furious. He stopped the training because he got so irritated… I think it was more with himself; it wasn’t the partner’s fault, but rather the frustration of not being able to perform as he wanted. And yes, he became aggressive, started raising his voice and so on. In the end, he left to calm down, but before that he shouted and raised his voice quite a lot. (Canary, Boxing, 26 years old, woman, our translation).

In Canary’s account, the practitioner’s frustration is directed primarily toward his own performance. The episode is marked by anger, raised voice, interruption of training, and temporary withdrawal from the situation. No reorganization of the experience is described within the account.

Doppo similarly describes frustration during kumite. Although he participates comfortably in other components of karate training, he reports difficulty striking his partners because he fears injuring them. During a fight with a practitioner of the same rank, repeated difficulty avoiding and landing attacks produces irritation and disappointment:

**Interviewee**: I have a colleague — the same rank as me — much younger than I am. When it started, with the hajime [command to begin the fight], he would already land a strike on me, and that kept irritating me. I wanted to land a strike as well, and that brought up a certain anger. I wanted to hit him but could not manage to do it, so I took about three strikes and he won the fight. But it wasn’t a loss of control, it was a… it was a disappointment with myself.

(...)

When it comes to kumite I always lack the strength to strike the other person, because I’m afraid of hurting them. So, kumite training sessions, all of them, are very challenging for me. (...) So much so that, kind of intuitively, I end up skipping the class — I find a way to arrange another commitment and things like that. (Doppo, Karate, 61 years old, man, our translation).

Doppo distinguishes his reaction from a loss of control and describes it instead as disappointment with himself. His fear of injuring others limits his engagement in kumite, and the continuing difficulty leads him to avoid these training sessions. In this case, the tension associated with fighting remains unresolved within his account and is followed by withdrawal from this component of practice.

Karla describes another disruptive response during training with a practitioner oriented toward competition. After being immobilized and facing submission, the training partner becomes distressed, cries, leaves the training area, and refuses Karla’s attempt to discuss the episode:

**Interviewee**: Especially for those who train to compete. (...) It already happened that I went there in the middle of training, the girl was there and I went to fight with her, and she lost. She realized she would not be able to handle it and that she was going to be submitted. She lost control. At first she came at me aggressively. (...) When I immobilized her, her next reaction was desperation. She stopped breathing properly and became desperate. She started crying, crying and crying. Then she stood up and went outside, and I went there to talk to her, but she did not want to talk to me. (Karla, BJJ, 34 years old, woman, our translation).

Karla’s account describes a transition from aggressive engagement to desperation and withdrawal. The training partner does not resume the interaction and declines subsequent dialogue, leaving the disruptive experience without an explicitly reported resolution.

Taken together, these accounts show that combative tension may be sustained, regulated, or experienced as disorganizing. Some participants describe increased attention, confidence, or capacity to remain engaged. Others describe anger, freezing, avoidance, interruption of training, emotional distress, or withdrawal from interaction. Within the psychic dimension, development is therefore not present in every disruptive situation. Rather, the accounts vary in whether practitioners become more able to experience combative pressure while maintaining their engagement in corporal fight.

### 4.2 Moral/spiritual dimension

The analysis identifies the Moral/Spiritual dimension as the practitioner’s positioning toward the integrity and well-being of both self and partner-opponent during combat. Across participants’ accounts, this dimension appears pre-reflectively as care or carelessness in close bodily engagement and in the regulation of combative intensity. It becomes especially visible when practitioners perceive excessive force, imminent injury, or conduct that exceeds the tacit limits of corporal fight. These situations intensify the psychic tension described above and bring the management of aggressiveness—understood here as the intensity of combative action—into focus.

This dimension depends on sensitivity to how combative pressure and bodily actions affect both participants. The accounts include situations in which this sensitivity is absent or insufficient, as well as situations in which practitioners regulate their actions to preserve the continuity of the fight. Canary describes an episode in which the intensity imposed by one training partner prompts a reaction from the other:

**Interviewee**: One is above the other and controls their strength, but the other… well, he gets carried away. I’m not exactly sure how to explain it, but it seems like he wants to assert himself. As he notices that the other is not using much force, he tries to outdo him and starts increasing the intensity, and the other gets startled and reacts. In the end, they end up getting hurt during training, and that is not the objective. (Canary, Boxing, 26 years old, female; our translation).

Canary describes a reciprocal escalation of force. Although one practitioner initially attempts to regulate the intensity, the other increases it in an effort to assert himself. The partner reacts, and both are injured. In her evaluation, the resulting injuries indicate that the interaction has departed from the purpose of training.

When asked how she identifies a loss of control, Canary further describes reduced technical organization, impulsive striking intended to hurt, and verbal confrontation:

**Interviewee**: (...) there is a certain level of aggressiveness when fighting. Maybe they become more… impulsive, lose some of their technique, and start striking in any way, just to land a strong blow and hurt the other. And, well, sometimes they end up insulting each other or arguing. (Canary, Boxing, 26 years old, female; our translation).

In Canary’s account, the regulation of force and technique distinguishes the pursuit of victory from conduct oriented toward injuring or offending the opponent. The loss of technical control, the intention to hurt, and the emergence of insults or arguments describe a disruption of the reciprocal structure of corporal fight.

Katara reports another configuration of this dimension involving unequal use of physical strength during mixed-gender training. She describes male partners who are substantially larger and stronger but increase their use of force after being technically challenged by a beginner woman. She interprets this reaction in relation to a “wounded ego”:

**Interviewee**: Most of the people I train with—men—are bigger than me. Regardless of whether they are more or less advanced, they are bigger and stronger, so I think it’s even kind of funny— it sort of hurts a man’s ego to see that a girl who is just starting is managing to “beat” him, right? “Beat” in quotation marks. And then they go ahead and start using full strength. There have been situations where I was underneath, with the other person on top, and the person using so much force (…) a man, using so much force, that I could not move. (Katara, BJJ, 26 years old, female; our translation).

Katara describes the excessive use of strength as restricting her movement and reducing her ability to participate in the roll. She contrasts these experiences with training alongside more experienced practitioners, stating, “I prefer to train with more advanced men, because they have a certain awareness” (Katara, BJJ, 26 years old, female; our translation). In her account, greater experience is associated with increased sensitivity to differences in size and strength and with a more appropriate regulation of force.

Gendered differences in physical strength recur in the accounts, particularly in situations in which male practitioners respond to being technically challenged or submitted by women. Karla describes an episode in which a partner increases his use of force after being submitted, resulting in a knee injury:

**Interviewee**: Imagine you and me rolling there. Look at my size, and then I go ahead and submit you in front of everyone. Some people do not know how to take that as a learning experience. They do not accept it. Then, when you start sparring again, they already come at you more forcefully. I’ve only had a few episodes like that—three, actually—and one of them really hurt me; I had to go to the hospital to have my knees checked, and it resulted in an injury. (…) But, overall, the coach always taught the guys to be respectful. So, in the end, he would always say, “there’s the issue of strength—you have to know how to handle it.” And then the guy who hurt me came over and talked to me, right? There was a whole situation. (Karla, BJJ, 34 years old, female; our translation).

Karla’s account connects the escalation of force with difficulty accepting submission and with the risk of injury. It also describes the coach’s explicit guidance regarding respect and the regulation of strength, which is also reflected in the accounts of other practitioners. The partner subsequently approaches Karla, indicating an attempt to address the episode, although the physical consequences remain.

Other accounts describe responses in which practitioners regulate intensity to preserve the continuity of training. Canary reports adjusting her strength and reducing the pace when a hesitant partner expresses concern about being struck:

**Interviewee**: Yes, there’s a girl from my team who… well, she has come up a few times to spar with me, but on many days, she does not want to get in. I think it’s because she spent some time away without training much, and I ended up becoming a bit more advanced than her. She would even tell me that directly (…) that she was hesitant to get in and all. (…) She would say, “Go easy, I don’t want you to hit me hard.” But, in the end, its training, and I would try to reassure her (…). I adjusted my strength. If I noticed that she was struggling, I would slow the pace a bit, and that was it — we continued well. (Canary, Boxing, 26 years old, female; our translation).

In this episode, Canary responds to her partner’s hesitation by adjusting both strength and pace. This regulation allows the partner to remain engaged and the sparring session to continue.

Mikasa similarly describes practitioners who recognize that they are striking with excessive force and modify their conduct. Their responses include changing partners, interrupting strikes before impact, and deliberately avoiding contact:

**Interviewee**: I’ve seen people starting to put more force into their strikes. (…) I’ve also seen people asking to switch sparring partners. These are people who realized what they were doing and tried to stop it in some way—either by changing partners, stopping the strike midway, or letting the strike pass through the air so as not to hit the other. (Mikasa, Boxing, 23 years old, female; our translation).

Across these accounts, the Moral/Spiritual dimension varies from reduced sensitivity or carelessness toward the partner-opponent to the practical regulation of force, intensity, and bodily action. Some situations are marked by escalation, technical disorganization, injury, or restricted participation. Others involve recognition of the partner’s limits and adjustments that allow training to continue. Personal development within this dimension appears as the progressive organization of a combative morality through which practitioners become more able to pursue victory while preserving the integrity and continued participation of those involved.

### 4.3 Bodily/technical dimension

The corporal/technical dimension occupies a primary position in participants’ accounts because corporal fight is lived through a bodily relation with the opponent. Psychic tension and moral/spiritual positioning become manifest through bodily engagement and require bodily responses within the fight. For beginners, the acquisition of combative techniques therefore emerges as a primary concern. This dimension concerns the progressive acquisition of modality-specific capacities—including attacks, counterattacks, defenses, posture, timing, distance, and movement—through which practitioners become able to perceive, respond to, and sustain the reciprocal dynamics of corporal fight. Across the analyzed accounts, the psychic and Moral/Spiritual Dimensions are experienced and enacted through the practitioner’s bodily possibilities.

Mikasa’s account illustrates this relationship. At the beginning of her boxing practice, she describes responding to sparring through hesitation and withdrawal, moving away from the opponent and lowering her guard:

**Interviewee**: Yes, I was very afraid. It wasn’t exactly fear of getting hit, it was more of a hesitation. Sometimes I would move away and end up lowering my guard—I tended to disengage from the fight. Little by little, I started to build more confidence, and I would actually go in with my strikes! (Mikasa, Boxing, 23 years old, female; our translation).

Her account describes a gradual movement from disengagement toward more active participation in the fight. She associates this change with increased confidence and with the acquisition of practical resources that allow her to strike, defend herself, and remain engaged under combative pressure:

**Interviewee**: As I learned new techniques, I felt more confident to go in, because I knew how to get out, or because I knew I could handle the other person’s punches. And gradually, over time, you feel that you are able to put into practice what you train. (Mikasa, Boxing, 23 years old, female; our translation).

In Mikasa’s account, technical learning expands the responses available within the fight. Knowing how to enter, exit, and withstand the opponent’s strikes reduces her tendency to withdraw and supports a more organized engagement with corporal fight.

Hatsumi similarly describes technical familiarity as changing how he responds to combative situations:

**Interviewee**: After some time training, if the situation happens, you know how to defend yourself. You either raise your guard more, or make some adjustment, anyway. But in the first week I did not even know what a guard was. I mean, I did not even know how to position my feet, or do anything. So, the more experience you have, the better you can defend yourself in these situations. In the end, experience brings skill, technical knowledge, and the ability to control different situations, so to speak. (Hatsumi, BJJ/MMA, 21 years old, male, our translation).

Hatsumi contrasts his initial inability to adopt a guard or position his feet with his later capacity to make defensive adjustments. In his account, increased familiarity with these basic actions provides a broader range of responses and a greater sense of control during combat.

**Interviewee**: You train with a blue belt, purple belt, brown belt... So, intimidated not really, but a bit... how can I say? A bit useless. (...) For example, in the beginning, (...) you start rolling and, without having any notion of anything, the person makes a movement and you simply get stuck, not knowing what to do, not knowing how to get out of there. This still happens to me, but in the beginning it was much worse. (Hatsumi, BJJ/MMA, 21 years old, male, our translation).

Rather than describing this experience primarily as intimidation, Hatsumi emphasizes his inability to recognize and respond to the opponent’s actions. Although this difficulty remains present, he retrospectively describes it as less pronounced than at the beginning of his practice.

He further reports that explanations received after being submitted provided specific possibilities for responding to similar situations:

**Interviewee**: In Jiu-Jitsu, for example, when a brown or purple belt applied that submission to me and I had no way out, afterward he explained three or four different ways to escape. So, when they teach you how to defend yourself in these situations, you end up acquiring knowledge. And, for example, with what happened with that guy, if he tried the same move now, in another sparring session, at a different moment, I could react better – raise my guard more, retreat more, or something like that. I think so, these experiences help. (Hatsumi, BJJ/MMA, 21 years old, male, our translation).

Hatsumi associates the instruction received from more experienced practitioners with the acquisition of specific defensive alternatives. He retrospectively anticipates that, when confronted with a similar action, he could respond through adjustments in guard, movement, or distance rather than remaining unable to act.

Choji describes a contrasting situation in which he lacks an organized technical response while being choked:

**Interviewee**: I was rolling with him, and he started choking me. I could no longer breathe and, at that moment, I started trying to get away from him in any possible way. (...) It was a situation in which I felt that I was losing a bit of control, I saw myself getting desperate and just wanted to get free, without thinking much about what I was doing. (Choji, BJJ, 27 years old, male, our translation).

Within this episode, the absence of an available technical response is accompanied by desperation and unplanned action. Choji describes attempting to escape in any possible way, without being able to organize his conduct in relation to the opponent’s action. The account does not describe a subsequent technical reorganization of this particular experience.

Canary also relates competitive tension to her limited experience and perceived technical preparation. Before her first competitive fight, she observes an opponent whom she considers physically larger and questions whether she entered competition before being sufficiently prepared:

**Interviewee**: I did not feel intimidated by my opponent, but it’s true that, while I was waiting to go up. (...) After all, we were in the same venue, and I saw her warming up. Physically, she was bigger than me, and at that moment I felt a bit unwell, because I thought that maybe I had made a mistake. In the end, I had little training time for the demands that boxing imposes. I think many more years are needed to master it, and I decided to compete with around 10 months of training. (Canary, Boxing, 26 years old, female, our translation).

Canary frames her hesitation through the perceived disparity between her limited training experience, the demands of boxing, and the physical characteristics of her opponent. Her account therefore presents competition as a situation in which perceived technical preparation and readiness become especially salient.

Across these accounts, the corporal/technical dimension ranges from restricted, improvised, or disorganized responses to a broader capacity to organize bodily action in relation to the opponent. Because corporal fight is lived through bodily engagement, technical possibilities provide the concrete means through which practitioners sustain combative tension, regulate their actions, and remain engaged with the partner-opponent. Technical learning does not eliminate combative tension, and the disruptive situations described do not uniformly result in development. When participants report change, it appears as an expansion of their embodied possibilities: maintaining a guard, adjusting distance, defending, escaping, striking, and remaining engaged in the fight. Personal development within this dimension is therefore identified as the progressive acquisition of practical resources that support the practitioner’s capacity to enter, respond to, and sustain corporal fight.

### 4.4 Integrative synthesis of the three dimensions

The analysis identifies personal development as a change in the practitioner’s lived relation to corporal fight —the manifestation termed corporal fight within the TPBC. At one pole, the beginner’s capacity to recognize, enter, and sustain the intentional structure of the fight remains limited or unstable. Combative tension appears accompanied by withdrawal, loss of control, reduced concern for the partner-opponent, or technically disorganized action. At the other pole, the practitioner becomes increasingly able to remain in the encounter, pursue victory, and preserve the reciprocal structure that constitutes it as corporal fight. In this sense, development appears as the progressive acquisition of the capacity to fight.

This change is expressed along three interrelated dimensions. In the psychic dimension, it consists in sustaining the tension inherent in combat without abandoning the intentional orientation of the fight. In the Moral/Spiritual Dimension, it consists in recognizing the partner-opponent and regulating one’s actions with concern for the integrity, wellbeing, and continued participation of those involved. In the bodily/technical dimension, it consists in incorporating the technical possibilities required to respond to the opponent and pursue victory through bodily action.

These dimensions do not represent separate or successive stages. They describe three axes along which the same developmental movement appears in different configurations across participants’ accounts. The prominence and articulation of each axis vary across the singular experiences examined. Nevertheless, the movement from a limited or unstable capacity to fight toward a more organized capacity to recognize, enter, and sustain corporal fight remains identifiable across the analysis (Figure 1).

**Figure 1:**
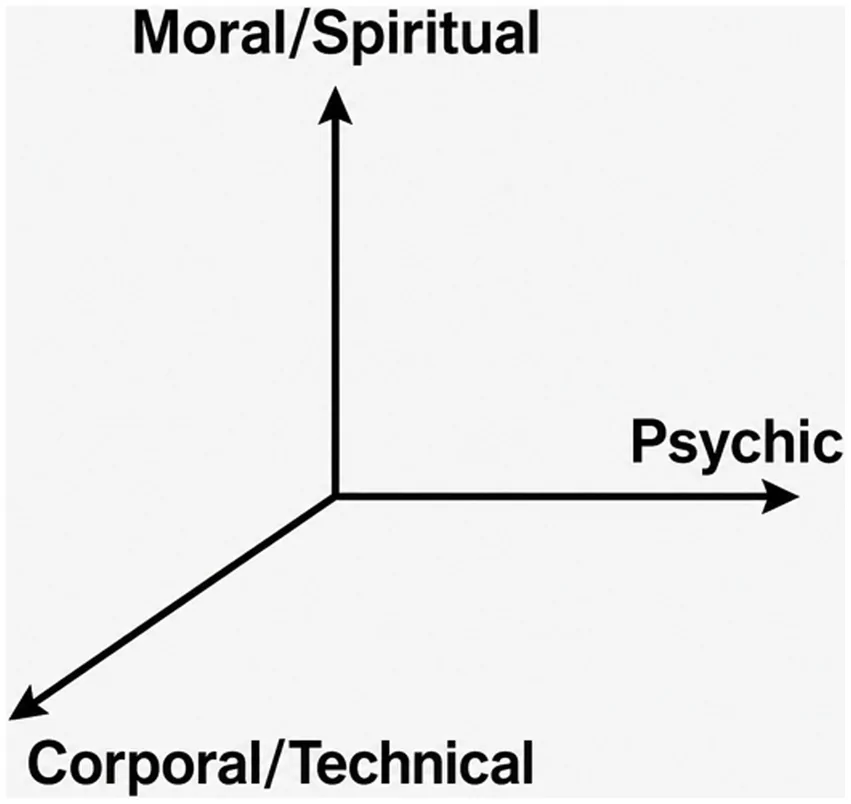
Three comprehensive dimensions of personal development in beginner MA&CS practitioners.

## 5 Discussion

The dimensions identified in this study constitute structural axes of the lived experience of combat. Their articulation allows us to comprehend how practitioners experience and respond to disruptive situations in training and competition. This perspective converges with phenomenological approaches to sport that emphasize the primacy of lived embodiment in understanding sporting practices (Allen-Collinson, 2009, 2011). The present analysis, however, adopts a classical phenomenological orientation grounded in the work of Edmund Husserl (1989) and Stein (1989, 1998).

In Ideas II, Husserl describes the person as a unity constituted through the articulation of bodily, psychic, and spiritual strata of experience (Husserl, 1989). This framework provides a basis for understanding the three dimensions identified in the present study as analytically distinguishable but inseparable within the unity of personal life. Stein further develops this account through her analyses of the person and empathy, showing how affective life, personal dispositions, and lived corporeality are intertwined within intersubjective experience (Stein, 1989, 1998). Empathy, understood as a non-primordial (not lived directly) experience of another subject’s experience, is particularly relevant to corporal fight because practitioners must perceive and respond to the partner-opponent as an embodied subject. Shared values and collectively established expectations of conduct also contribute to organizing this interaction (Husserl, 1989). Corporal fight is therefore not merely an encounter between individual psychological states, but a bodily and intersubjective relation jointly sustained by those involved.

In Ales Bello’s interpretation of Stein’s work, the bodily, psychic, and spiritual strata correspond to structural levels through which personal experience becomes manifest, while their unity is realized in the concrete dynamics of lived life (Ales Bello, 2005, 2010). From this perspective, the Psychic, Moral/Spiritual, and Corporal/Technical dimensions identified in participants’ accounts can be understood as interrelated manifestations of the lived experience of combat. Although they are distinguished analytically, they are concretely unified within combative interaction. Because corporal fight is lived through corporeal engagement with the partner-opponent, corporeality constitutes the concrete medium through which psychic tension, Moral/Spiritual positioning, and technical action become manifest.

### 5.1 Combative tension and the psychic dimension

One of the central findings concerns the tension inherent in corporal fight, even in the absence of hostility or interpersonal conflict. Participants describe intimidation, frustration, fear, and mental blockage, particularly when facing more experienced partners or the uncertainty associated with competition. These experiences indicate that combative tension does not arise only when the reciprocal structure of the fight is disrupted; it is already present in the bodily confrontation through which corporal fight is constituted.

These findings are consistent with previous research on emotional experiences in combat sports, in which anxiety, fear, and demands for emotional regulation are frequently reported, particularly during the early stages of practice (Chen et al., 2020; Kooi, 2020). The present analysis, however, suggests that these affective experiences should not be understood solely as individual psychological states requiring regulation. They also arise from the intersubjective structure of bodily confrontation, in which each practitioner is exposed to the actions, abilities, and intentions of the partner-opponent (Krabben et al., 2019). The Psychic dimension therefore concerns not the elimination of tension, but the practitioner’s capacity to experience and respond to it while remaining engaged in corporal fight.

This interpretation complements previous phenomenological analyses of combat. Barreira (2019b), for example, shows that practitioners may not experience combat sports as violent despite the exchange of strikes, because the encounter is sustained by a reciprocal disposition to fight. The beginners’ accounts extend this interpretation by showing that learning to inhabit this intersubjective field is neither immediate nor uniform. For some practitioners, combative tension is followed by increased attention, confidence, and a greater capacity to remain in the encounter. For others, it remains associated with freezing, emotional disorganization, interruption of training, or avoidance of fighting. Disruptive situations therefore do not necessarily produce psychic development. Rather, development appears when practitioners progressively become able to sustain combative pressure without either reducing the encounter to aggression or withdrawing from its combative demands.

### 5.2 The moral/spiritual dimension of the corporal fight

A second contribution of this study concerns the Moral/Spiritual dimension of corporal fight. The findings identify this dimension as a practical and embodied sensitivity to how one’s actions affect the integrity, well-being, and continued participation of the partner-opponent. It becomes manifest in the modulation of force and pace, the perception that a partner is under excessive pressure, and the adjustment or interruption of one’s actions when necessary. However, this sensitivity is not uniformly present across the accounts. Episodes of escalating intensity, restricted participation, and injury show that Moral/Spiritual regulation may remain insufficient or break down during combative interaction.

These findings contribute to recurring debates on aggressiveness and moral regulation in MA&CS. Previous studies have examined whether participation reduces or increases aggressiveness, but the evidence remains inconclusive (Lafuente et al., 2021; Xu et al., 2023). Some studies associate practice with outcomes such as greater self-control and personal development (Theeboom, 2012; Wen et al., 2020), whereas others find no clear relationship between MA&CS participation and traits related to aggressiveness (Lafuente, 2024; Lafuente et al., 2021).

The present study approaches this issue from a different analytical level. Rather than treating aggressiveness primarily as an individual personality trait, it examines how combative intensity is perceived and regulated within the encounter itself. In this analysis, aggressiveness refers to the intensity of combative action and should not be equated with hostility. Corporal fight requires practitioners to pursue victory while remaining sensitive to the partner-opponent’s bodily condition and to the tacit limits that preserve the reciprocal structure of the fight. This practical orientation corresponds to what Barreira (2017c) describes as a “sensitive norm”: a pre-reflective regulation through which practitioners modulate their actions in relation to the other. The Moral/Spiritual Dimension is therefore not an abstract moral rule applied after the action; it is enacted corporeally through the control of force, timing, pace, and technical execution.

The accounts of female practitioners make particularly visible how this regulation is shaped by the social context of training. Katara and Karla describe situations in which male partners increased their use of force after being technically challenged or submitted by women. These reactions restricted participation and, in one case, resulted in injury. Although the present sample does not support a systematic comparison of gendered experiences, these accounts indicate that differences in size and strength, gender expectations, and attempts at self-assertion may affect how force is employed within mixed training. Similar tensions have been identified in sociological research on mixed-gender martial arts practice, particularly regarding gender norms and the practical legitimacy of using force against women (Channon, 2013; Channon and Jennings, 2013). Research on women’s participation in MA&CS likewise shows that combative interaction may expose tensions between broader cultural expectations and the specific demands of training, requiring situational rearrangements of the norms governing bodily contact and opposition (Figueiredo et al., 2021).

At the same time, participants describe partners who reduce their pace, adjust their strength, interrupt strikes before impact, or request a change of partner after recognizing that the interaction has become excessive. Coaches also appear in the accounts as figures who explicitly frame respect and the appropriate management of strength. Moral/Spiritual regulation is therefore not constituted solely as an individual achievement; it is relationally organized through peer conduct, instructor guidance, and the norms of the training environment. Recent scholarship similarly emphasizes that the health-promoting, inclusive, and developmental possibilities of combat sports must be considered alongside their risks and supported by ethically responsible coaching, safe training environments, protection, and sport-integrity practices (Ciaccioni et al., 2025b; Ciaccioni et al., 2025a; Ciaccioni et al., 2026).

The findings therefore do not support the assumption that participation in disruptive combat situations automatically produces moral development. Such situations expose the need to regulate intensity, but their outcomes vary according to how practitioners, partners, and coaches respond. Development within the Moral/Spiritual dimension appears when practitioners become progressively more capable of perceiving the partner-opponent’s condition and modulating their own actions without abandoning the intention to fight. Conversely, failures of sensitivity and regulation may lead to escalating force, injury, withdrawal, or a disruption of the reciprocal structure that distinguishes corporal fight from brawl.

### 5.3 Technical learning and the transformation of the combat experience

The Corporal/Technical dimension occupies a primary position in the developmental process because corporal fight is constituted and lived through bodily engagement with the partner-opponent. Psychic tension and Moral/Spiritual positioning do not occur independently of corporeality; they become concrete through posture, movement, distance, timing, force modulation, attacks, counterattacks, and defenses. Technical learning should therefore not be understood merely as the acquisition of motor skills. It involves the progressive incorporation of a bodily *savoir-faire* through which practitioners perceive new possibilities for action and begin to inhabit the combative situation differently.

Across participants’ accounts, the acquisition of technical knowledge reorganizes the perceptual and practical field of combat. As defensive and offensive possibilities become incorporated into the lived body, practitioners may become more capable of remaining engaged under pressure, recognizing the opponent’s actions, and responding in an organized rather than improvised manner. Phenomenological analyses of beginners across different fighting modalities similarly describe learning to fight as a transformation of the perceptual field through which combative actions become increasingly intelligible and actionable (Telles et al., 2018). Technical development thus concerns both what practitioners are able to do and how the combat situation itself becomes available to perception and action.

The centrality of embodied technical learning is also supported by sociological and phenomenological analyses of combat sports. Wacquant (2006), for example, describes boxing training as a process through which practical knowledge is progressively incorporated, transforming the practitioner’s bodily perception of fighting. From an ethnomethodological perspective, entering combat likewise requires the acquisition of specific practical dispositions. Sánchez García (2008) illustrates this process through the case of a beginner boxer who abandons training after being struck and remains unable to bodily sustain the fighting situation. Such episodes show that combative pressure may initially disorganize the practitioner’s practical field. They also indicate that disruptive experiences do not automatically result in technical development: bodily possibilities must be progressively incorporated through continued practice for the practitioner to respond differently to comparable situations.

In the present study, this process remains closely articulated with the Psychic and Moral/Spiritual Dimensions. As practitioners expand their technical repertoire, they may become more capable of sustaining combative tension and regulating the intensity of their actions in relation to the partner-opponent. However, understanding that force should be regulated does not mean that beginners can immediately regulate it in action. Combative pressure may lead either to excessive force or to excessive restraint motivated by fear of injuring the partner. These difficulties illustrate the ongoing reorganization of practical dispositions within the combative situation (Sánchez García, 2008).

Technical learning may provide the embodied resources through which these dimensions become more stably articulated. The practitioner must not only recognize the need to protect the partner-opponent, but also possess the bodily means to control distance, timing, force, and technical execution while continuing to pursue the aims of the corporal fight. Conversely, technical competence alone does not ensure Moral/Spiritual regulation or a developmental outcome. Expanded bodily possibilities may be used with greater sensitivity and control, but they may also support excessive force when the reciprocal orientation of corporal fight is not preserved.

The phenomenological primacy of the Corporal/Technical dimension should therefore not be understood as reducing personal development to technical proficiency. Rather, it indicates that Psychic and Moral/Spiritual changes must be enacted through corporeality. When development is described, it appears as an expansion of embodied possibilities that allows practitioners to remain under pressure, act effectively, and regulate their actions while preserving the partner-opponent’s continued participation. Technical skills thus provide a concrete condition for sustaining corporal fight as a bodily and reciprocal encounter, without by itself guaranteeing that such an encounter will be developmental.

### 5.4 Competition as a particularly tension-laden experience

Competition emerged in participants’ accounts as an intensification of the dynamics described across the three dimensions. First competitive experiences were frequently associated with fear, anxiety, and uncertainty, suggesting that competition may constitute a particularly demanding moment in the early process of learning to fight. In this context, practitioners must sustain heightened combative tension while mobilizing technical resources and regulating their actions toward an unfamiliar opponent.

Competitive corporal fight also presents specific experiential demands. For beginners, competition involves facing an unknown opponent and more fully assuming an orientation toward victory, with fewer opportunities to negotiate or reduce intensity than are usually available in learning and training contexts. Such contexts may include forms of engagement situated near the boundary between fighting and play fighting, in which intensity can be adjusted according to the partner’s experience and responses (Krabben et al., 2019). By contrast, competition is organized through a hierarchy of motivations in which the achievement of sporting results becomes a primary determinant of conduct (Witkowski et al., 2013).

Under these conditions, combative tension may intensify before the encounter itself begins. Phenomenological analyses indicate that entering combat involves anticipatory bodily adjustments and an increasing attentional orientation toward the opponent, through which the fight begins to be constituted before the first exchange of actions (Telles et al., 2018). In competition, this anticipatory process occurs under heightened pressure and places greater demands on the practitioner’s capacity to remain engaged, respond technically, and preserve the reciprocal orientation of corporal fight. This intensification does not imply an inevitable movement toward hostility, but it may expose the limits of the practitioner’s current psychic, Moral/Spiritual, and Corporal/Technical resources.

Previous research has emphasized the possible formative significance of disruptive competitive experiences. Santos et al. (2025), for example, report that masters from different countries interpret such situations as opportunities for developing emotional control and resilience. The present findings complement this perspective by showing how beginner practitioners retrospectively describe their first encounters with competitive pressure. Some accounts include increased confidence and recognition of one’s capacity to fight, whereas others remain marked by fear, perceived technical insufficiency, or uncertainty regarding readiness to compete.

Competition should therefore not be treated as inherently developmental. Rather, it constitutes a particularly intense context in which the practitioner’s existing resources and limitations become more visible. Its developmental relevance may depend on adequate technical preparation, progressive exposure to competitive demands, appropriate opponent selection, and guidance capable of preserving both physical and emotional safety. Within this perspective, competition readiness involves more than willingness to participate; it requires sufficient capacity to sustain combative pressure, respond effectively, and remain within the reciprocal structure of corporal fight.

### 5.5 Rethinking personal development in MA&CS

Taken together, the findings contribute to broader debates on personal development in MA&CS. Much of the literature examines development through psychological constructs such as self-control, emotional regulation, or changes in aggression-related traits (Chen et al., 2020; Xu et al., 2023). Although these approaches provide important insights, they may overlook the experiential specificity of learning to fight and the bodily and relational structure through which development is lived in MA&CS.

From the classical phenomenological perspective adopted in this study, personal development can be understood as a possible reorganization of the practitioner’s capacity to enter, respond to, and sustain corporal fight. This process involves becoming more able to experience combative tension without withdrawing or losing control, to remain sensitive to the integrity and continued participation of the partner-opponent, and to mobilize the embodied technical resources required by the encounter. Because corporal fight is constituted as a bodily relation, the Corporal/Technical dimension provides the concrete field through which changes in the Psychic and Moral/Spiritual dimensions are enacted.

The three dimensions should not be understood as separate outcomes or successive developmental stages. Rather, they describe interdependent aspects of a change in how practitioners inhabit the combative situation. When development is reported, it appears as an expansion and organization of embodied possibilities through which practitioners become increasingly capable of remaining under pressure, acting effectively, and preserving the reciprocal structure of the corporal fight. Personal development therefore does not primarily consist in reducing aggressiveness or modifying stable personality traits. It concerns a transformation in the practitioner’s lived relation to combat.

This transformation, however, is neither uniform nor guaranteed. The disruptive situations described in the interviews expose the limits of practitioners’ existing Psychic, Moral/Spiritual, and Corporal/Technical resources, but they do not necessarily produce their reorganization. Some accounts involve increased confidence, technical competence, and practical sensitivity, whereas others remain marked by avoidance, emotional disorganization, excessive force, injury, or withdrawal. Disruption should therefore be understood as a possible condition for experiential reorganization rather than as an inherently developmental event.

This interpretation converges with recent phenomenological analyses suggesting that the developmental significance of combat sports lies in the demand to sustain combative tension while continuing to recognize the opponent as a partner in the encounter (Barreira et al., 2025). The contribution of the present study is to specify how this process becomes manifest among beginners through the interrelation of the Psychic, Moral/Spiritual, and Corporal/Technical dimensions. Personal development in MA&CS thus appears as the progressively organized capacity to inhabit corporal fight as an embodied, reciprocal, and technically mediated relation.

### 5.6 Contributions and implications

This study contributes to the MA&CS literature by providing an empirically grounded phenomenological account of how beginner practitioners experience processes of personal development in relation to disruptive situations. By focusing on lived experience, it addresses a gap in research that often privileges external outcomes while overlooking the experiential dynamics through which practitioners learn to fight. The findings also advance a psychology of MA&CS by identifying three interdependent dimensions of this process—the Psychic, Moral/Spiritual, and Corporal/Technical dimensions—and by showing that their unity is concretely realized through bodily engagement with the partner-opponent. Together, these dimensions provide a conceptual framework for understanding how practitioners progressively become able to enter, respond to, and sustain corporal fight.

The findings also have practical implications for instructors working with beginners. They do not support the assumption that exposure to disruptive combat situations is inherently developmental. Rather, the developmental possibilities of such experiences appear to depend on how combative pressure is pedagogically and relationally organized. Recent scholarship similarly emphasizes that the health-promoting, inclusive, and developmental potential of MA&CS depends on structured instruction, safe training environments, ethically responsible coaching, injury prevention, and the careful management of the risks inherent to combative practice (Ciaccioni et al., 2025b; Ciaccioni et al., 2025a; Ciaccioni et al., 2026).

From the perspective of the Psychic dimension, beginners may benefit from gradual exposure to combative pressure rather than immediate immersion in highly intense sparring or competition. Fear, freezing, avoidance, and emotional disorganization should not necessarily be interpreted as failures of motivation; they may indicate that the demands of the situation exceed the practitioner’s current capacity to remain engaged. Instructors can therefore support emotional safety by progressively increasing the unpredictability and intensity of combat, clearly communicating the aims of each exercise, and attending to persistent withdrawal or avoidance.

The Corporal/Technical dimension indicates that beginners require sufficient defensive and offensive resources before being exposed to situations in which intensity and uncertainty are substantially increased. Technical learning provides the embodied possibilities through which practitioners can maintain posture, regulate distance, defend themselves, escape, and respond without relying on improvised or disorganized action. Competition readiness should therefore not be defined solely by training duration or willingness to compete. It also involves the practitioner’s capacity to sustain pressure, respond technically, and regulate bodily action within the reciprocal structure of corporal fight.

The Moral/Spiritual dimension highlights the importance of partner selection, force regulation, and explicit norms of conduct. Coaches should consider differences in experience, size, strength, and technical ability when pairing beginners and should monitor situations in which gendered expectations or unequal physical conditions affect how force is employed. Communication between partners regarding pace and intensity, interruption of actions when control is lost, and pedagogical intervention after episodes of escalation may help preserve both physical safety and continued participation. The accounts also indicate that peers are not merely external influences on individual development: through their sensitivity, restraint, and willingness to adjust, they participate directly in sustaining the combative relationship.

These implications situate personal development within a broader training culture. Coaches, partners, and institutional norms shape whether disruptive situations are followed by learning and reorganization or by injury, avoidance, hostility, and withdrawal. Training environments that normalize excessive force, disregard fear, or treat unequal interactions as tests of personal toughness may intensify negative trajectories. Conversely, environments that combine graded exposure, technical preparation, relational responsibility, and opportunities to reflect on disruptive episodes may provide more favorable conditions for safe and sustainable participation. This interpretation is consistent with recent calls to translate MA&CS research into evidence-informed educational and health-promoting practices without disregarding the conditions required to minimize adverse outcomes (Ciaccioni et al., 2025b; Ciaccioni et al., 2025a; Ciaccioni et al., 2026).

Together, the three dimensions provide a conceptual basis for the development of pedagogical resources, instructor education, and training programs focused on beginner participation. One practical application of this framework is the ICM UNESCO Martial Arts Open School São Paulo 2025, conducted in Ribeirão Preto, Brazil, under the auspices of the ICM-UNESCO. The initiative is presented here as an example of knowledge translation; its effectiveness is not evaluated in the present study.

The findings also offer a possible explanation for inconsistencies in research on aggression in martial arts. When aggressiveness is examined primarily as an individual trait detached from the structure of the combative encounter, important dimensions of practice may remain obscured. From the present phenomenological perspective, combative intensity is not equivalent to hostility. Its experiential meaning depends on how action is intentionally and relationally organized. Practical norms, technical control, and recognition of the partner-opponent sustain corporal fight, whereas their disruption may allow the encounter to shift toward hostility or brawl. Recent scholarship similarly emphasizes the importance of conceptual clarity in distinguishing regulated combat from harmful aggression and of training cultures that explicitly cultivate self-regulation, relational ethics, and safety norms (Ciaccioni et al., 2026).

The inclusion of interviews conducted in Brazil and Spain broadens the range of experiences considered in this analysis. However, the study does not establish that combative practices are experienced identically across these contexts. Cultural norms may shape instructor authority, relationships between training partners, expectations regarding force and competition, gendered interactions, and the meanings attributed to endurance, fear, and self-control. Because the study was not designed as a comparison between countries, it cannot distinguish cultural influences from differences related to modality, academy, or individual trajectory. Future comparative research could examine how the three dimensions are expressed and organized within different cultural and institutional settings.

### 5.7 Limitations

The findings should be interpreted in light of the study’s retrospective qualitative design. This design provides access to how practitioners currently understand and interpret their experiences, but it does not establish that disruptive events produce personal development. Participants’ accounts may be shaped by memory, subsequent experiences, and later reinterpretations of the situations described. The absence of longitudinal observation also prevents the study from examining how the reported processes unfold over time. The findings should therefore be understood as phenomenological descriptions of lived and retrospectively interpreted processes rather than as evidence of causal change.

The sample comprises 17 practitioners from eight MA&CS disciplines interviewed in Brazil and Spain. Although this diversity broadens the range of combative experiences represented, it may obscure discipline-specific and culturally situated differences. The study was not designed to compare countries or modalities, and the transferability of its findings to other populations, training environments, and cultural contexts is therefore limited. The sample also does not support a systematic analysis of gendered experiences or unequal training interactions, despite the relevance of these issues in several participants’ accounts. Future studies should examine cultural, disciplinary, and gender-related variations using larger and more specifically designed samples.

Finally, empirical-phenomenological analysis depends on researcher expertise and interpretive judgment. The first author performed the final classification of beginner practitioners and conducted the eidetic and intersubjective reductions without an independent parallel classification. Shared methodological training, peer discussion, and collective examination of the psychological reductions supported analytical consistency, but these procedures do not eliminate the influence of the first author’s interpretation. A degree of circularity may also remain between the experiential classification of beginners and findings concerning fear, uncertainty, and technical difficulty, even though none of these manifestations was treated as a necessary or sufficient criterion for inclusion. These methodological characteristics may limit the reproducibility of the classification and should be considered when interpreting the proposed dimensions.

## 6 Conclusion

The findings indicate that disruptive experiences constitute privileged moments in which the structure of corporal fight becomes particularly visible to beginner practitioners. These situations mobilize three interdependent dimensions of experience: the Psychic dimension, expressed in the tension inherent to bodily confrontation; the Moral/Spiritual dimension, expressed in the regulation of combative intensity in relation to the partner-opponent; and the Corporal/Technical dimension, through which practitioners acquire the embodied resources required to enter, respond to, and sustain the fight. When development is described, it appears as a progressive reorganization of these dimensions, through which practitioners become more capable of inhabiting the combative situation in a stable, effective, and relationally sensitive manner.

Disruptive experiences, however, are not inherently developmental. Participants’ accounts include increased confidence, technical competence, and practical sensitivity, but also avoidance, emotional disorganization, excessive force, injury, and withdrawal. Their developmental significance therefore depends on the practitioner’s existing resources and on the pedagogical and relational organization of practice, including instructor guidance, peer conduct, partner selection, and regulation of training intensity.

These findings contribute to understanding MA&CS as contexts in which technical learning, the regulation of aggressiveness, and recognition of the other are intertwined within the dynamics of fighting. Rather than reducing personal development to changes in isolated psychological traits, the analysis suggests that it may involve the constitution of a new experiential horizon in which practitioners become increasingly able to sustain combative engagement without dissolving its embodied and intersubjective structure. The study thus contributes conceptual and empirical foundations for a phenomenologically grounded Psychology of Martial Arts and Combat Sports attentive to transformations in corporeality, perception, and Moral/Spiritual sensitivity within the experience of corporal fight.

## Data Availability

The datasets presented in this study can be found in online repositories. The names of the repository/repositories and accession number(s) can be found in the article/Supplementary material.

## Footnotes

1 The development of this theory is described in detail by Barreira et al. (2025).
