# Supplementary material for: Becoming a fighter: phenomenological dimensions of personal development in beginner martial arts and combat sports practitioners
Source: Front Psychol. 2026 Sep 18;17:1910206. doi: 10.3389/fpsyg.2026.1910206 (PMC13630589; doi:10.3389/fpsyg.2026.1910206)
Supplement: Supplementary file 1 [file Supplementary_file_1.docx]

**Supplementary Materials**

# Research Interview Guide: Martial Arts, Subjectivity, and Control

# 1. Preliminary Identification and Demographics

- Name:
- Education (Highest Degree/Current Study):
- Occupation:
- Age:
- Discipline (Modalidade):
- Rank/Belt Level (Graduação):
- Years of Practice:

# 2. Topic 1: Development and Self-Control (Student/Practitioner Perspective)

## The Practitioner’s Journey

- Describe your entry into this martial art. How did you begin practicing this discipline?
- Since you started, how would you describe your experience of training and learning within this art?
- Can you identify internal changes in yourself since you began your practice?
  - Describe how you have changed over time through this practice.
  - [Probe: How did you perceive these changes happening? Were there specific physical markers, such as changes in breath or tension, or emotional markers, such as increased patience or a different response to stress?]

## Delicate Situations and Loss of Control

- Describe a situation during practice that felt delicate, difficult, tense, or conflict-ridden.
  - How did you perceive the situation at the time?
  - What was it like to experience that moment as it was happening?
  - Reflecting on it now, how do you evaluate that experience? Have you encountered similar situations since then?
- Describe a situation where you experienced a loss of control.
  - [Note to Interviewer: Loss of control may manifest as emotional outbursts, physical aggression, or a total breakdown of technical form.]
  - Do you perceive modifications in your way of acting after experiencing this loss of control?
- Describe a time you witnessed another practitioner lose control.
  - Do you perceive changes in your own behavior or mindset after witnessing this?
- Describe a moment where you felt you were on the verge of losing control but did not.
  - Do you perceive changes in your way of acting after experiencing a "near-loss"?
- Describe a time you witnessed someone else in a state of near loss of control.
  - Do you perceive changes in yourself after witnessing such a moment?

## Intimidation

- Describe a time you felt intimidated while practicing, sparring, or fighting.
  - How did this experience modify your way of acting afterward?
  - [Probe: Does this influence your willingness to spar with certain partners or your confidence in your technique?]
- Have you ever noticed someone else appearing intimidated while practicing with you?
  - How did witnessing their intimidation affect your own behavior or approach to the training?

## Concluding Reflection

- Reflecting on our conversation, are there any other internal or behavioral changes you can recall or perceive that we haven't discussed yet?

# 3. Topic 2: Teaching, Mentorship, and Student Observation (Instructor Perspective)

*Note: This section is intended specifically for those in teaching, coaching, or master-level roles.*

## Observing Student Understanding and Growth

- How do you perceive a student's understanding of this martial art?
- How do you perceive a student's growth within the discipline?
  - In your view, how does this growth occur?
  - What specific indicators do you use to recognize that this growth is happening?

## Observations of Control in Combat

- Have you observed your students or practitioners experiencing moments of losing control during combat practices or sparring?
  - [Note to Interviewer: Explore the differences between these occurrences in a tournament setting versus a classroom/dojo setting.]
- In your observation, do these experiences cause a change in the student's way of fighting?
- Do these experiences modify the student’s overall way of being?
- What is the importance of these experiences in a practitioner's development?
  - [Note to interviewer: Do you view these moments as a pedagogical tool, a safety risk, or perhaps a necessary rite of passage?]

**Observations of Near Loss of Control**

- Have you observed your students or practitioners experiencing near-loss of control situations during combat or sparring?
  - [Note to Interviewer: Explore the differences between these occurrences in a tournament setting versus a classroom/dojo setting.]
- Do you perceive these "near-miss" experiences as causing a change in how the student fights?
- Do these experiences modify the student’s "way of being" outside of combat?
- What do you consider to be the importance of these near-loss experiences?

# 4. Topic 3: Gender Dynamics and the Experience of Women in Martial Arts

## Entry and Environment

- How did you first enter the world of martial arts?
- Describe the moment you joined a mixed-gender training environment.
  - How would you describe your training group or team? Did you join by recommendation, through friends, or as a specific personal choice?

## Gender-Specific Experiences

- While practicing your martial art, have you experienced situations that you attribute specifically to the fact that you are a woman? Please describe these moments in detail.
- Do you recall any instances where training was conducted differently for men than it was for women?
- Have you ever experienced a situation during training that caused you discomfort, disadvantage, or embarrassment because you are a woman?
- Conversely, have you ever experienced a situation during training that resulted in a positive sensation or an advantage because you are a woman?

## Comparative Training Dynamics

- In your view, are there differences between training with men versus training with women?
  - [Note to interviewer: You must prompt for observations regarding Rank, Age, Weight, Strength, and Technical Quality.]
  - Can you provide a specific example or situation that illustrates these differences?
- Describe your relationship with your male and female training partners, highlighting any situations that stand out to you.

## Evaluation of Treatment

- Have you ever perceived a difference in how you are treated compared to other practitioners?
- How did you perceive this difference, and do you evaluate that treatment as appropriate or inappropriate?

# 5. Topic 4: Exemplary and Reproachable Practices

## Ethical Conduct

- Throughout your experience in this discipline, describe a delicate, difficult, or tense situation where you felt the conduct of a teacher or a senior student was exemplary in handling the moment.
- Conversely, describe any actions or conduct in similar situations that you would consider reproachable.

# 6. Topic 5: Political and Ideological Inclinations

## Institutional and Personal Perception

- Do you consider your specific martial art discipline to have any established political inclinations or a particular institutional reputation?
- Beyond institutional reputation, do you consider the discipline to have any political-ideological tendencies?
  - [Note to interviewer: For example, do you perceive the community or the art's values as leaning more toward the left or the right on the ideological spectrum? How does this manifest in the worldviews or values shared within the practice?
